# Evidence of nutrient translocation in response to smoke exposure by the East African ant acacia, *Vachellia drepanolobium*

**DOI:** 10.1002/ece3.8244

**Published:** 2021-10-28

**Authors:** Richard Rabideau‐Childers, Katherine I. W. Angier, Brendan Z. M. Dean, Meghan Blumstein, Walker S. Darling, Annina Kennedy‐Yoon, Clayton H. Ziemke, Christian A. Perez‐Martinez, Donghao Wu, Wenqing Ye, Inam Yekwayo, Duncan M. Kimuyu, Dino J. Martins, Naomi E. Pierce

**Affiliations:** ^1^ Department of Organismic and Evolutionary Biology and Museum of Comparative Zoology Harvard University Cambridge Massachusetts USA; ^2^ Department of Civil and Environmental Engineering MIT Cambridge Massachusetts USA; ^3^ Division of Biological Sciences University of Missouri Columbia Missouri USA; ^4^ College of Life Sciences Zhejiang University Hangzhou China; ^5^ School of Biology and Environmental Sciences University of Mpumalanga Mbombela South Africa; ^6^ Department of Natural Resources Karatina University Karatina Kenya; ^7^ Mpala Research Centre Nanyuki Kenya; ^8^ Department of Ecology and Evolutionary Biology Princeton University Princeton New Jersey USA

**Keywords:** fire, Kenya, root‐shoot ratio, savanna, transport, volatile

## Abstract

1. Fire is a major selective force on arid grassland communities, favoring traits such as the smoke‐induced seed germination response seen in a wide variety of plant species. However, little is known about the relevance of smoke as a cue for plants beyond the seedling stage.
2. We exposed a fire‐adapted savanna tree, *Vachellia* (=*Acacia*) *drepanolobium*, to smoke and compared nutrient concentrations in leaf and root tissues to unexposed controls. Experiments were performed on three age cohorts: 2‐year‐old, 9‐month‐old, and 3‐month‐old plants.
3. For the 2‐year‐old plants exposed to smoke, carbon and nitrogen concentrations were lower in the leaves and higher in the roots than controls. Less pronounced trends were found for boron and magnesium.
4. In contrast, smoke‐exposed 3‐month‐old plants had lower root nitrogen concentrations than controls. No significant differences were found in the 9‐month‐old plants, and no significant shifts in other nutrient concentrations were observed between plant tissues for any of the three age cohorts.
5. *Synthesis*: Our findings are consistent with smoke‐induced translocation of nutrients from leaves to roots in 2‐year‐old *V*. *drepanolobium*. This could represent a novel form of fire adaptation, with variation over the course of plant development. The translocation differences between age cohorts highlight the need to investigate smoke response in older plants of other species. Accounting for this adaptation could better inform our understanding of savanna community structure and nutrient flows under fire regimes altered by anthropogenic land use and climate change.

## Graphical abstract

We exposed a fire‐adapted savanna tree, *Vachellia* (=*Acacia*) drepanolobium, to smoke and compared nutrient concentrations in leaf and root tissues to unexposed controls. Our findings suggest trees may detect smoke and respond by translocating nutrients from leaves to roots, a potential mechanism for improving survival and post‐fire resprouting.

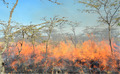

## 1 INTRODUCTION

In arid grassland ecosystems, fire is a major selective pressure on plant traits (reviewed in ref. Bond & Keeley, 2005), influencing community structure (Axelrod, 1985; Sankaran et al., 2008; Van Langevelde et al., 2003) and nutrient cycling (Anderson et al., 2007; Cech et al., 2008; Knapp & Seastedt, 1986). The key to the maintenance of the savanna biome state is that fires can act as a balancing force between trees and grasses (Sankaran et al., 2005; Staver et al., 2011). While there is a long history of human additions to natural patterns of burning, such as for hunting, grazing livestock, and planting crops (Marchant, 2010; Martins de Melo & Saito, 2012; Silva et al., 2006), in more recent decades, fire suppression and increased grazing are shifting the balance in favor of trees (Stevens et al., 2016; Venter et al., 2018). This woody encroachment is a land management challenge, resulting in reduced forage for livestock and augmentation of tick populations (Negasa et al., 2014) and shifting landscape‐scale biogeochemistry and hydrology (Asner et al., 2004). Both the balance of vegetation types (Hagos & Smit, 2005) and fire frequency (Jensen et al., 2001; Wan et al., 2001) influence savanna nutrient dynamics, with implications for ecological stability and human use (Venter et al., 2017). Understanding the impact of fire on these ecosystems is the key to management recommendations.

**FIGURE 1:**
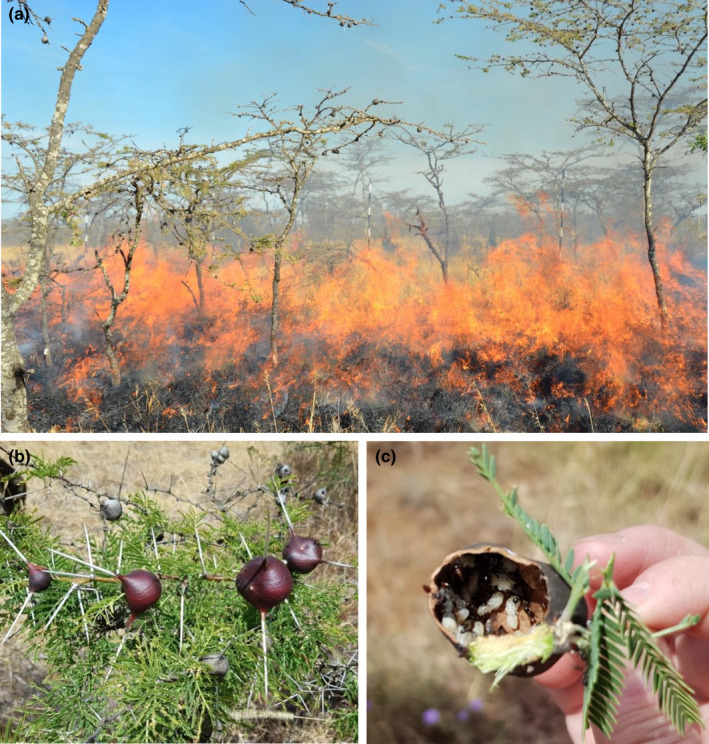
The ant‐acacia symbiosis. (a) *Vachellia drepanolobium* being burned in a controlled fire in the Kenya Long‐Term Exclosure Experiment plots, at the Mpala Research Centre in Laikipia, Kenya. (b) The swollen stipular thorns form ‘domatia’ that house the defensive ant symbionts and provide further defense against herbivores. (c) An opened domatium. At Mpala, four symbiotic ant species coexist in the landscape (though rarely within individual trees): *Crematogaster mimosae*, *Crematogaster nigriceps*, *Tetratponera penzigi*, and *Crematogaster sjostedti*. Trees used for this experiment at the Harvard Museum of Comparative Zoology greenhouse did not house ants. Photos by (a) Duncan Kimuyu and (b, c) Brendan Dean

We examine a potential fire response in one species contributing to woody encroachment (Kimaro & Treydte, 2021), the East African Whistling Thorn Acacia, *Vachellia (*=*Acacia) drepanolobium* (Figure 1). *Vachellia drepanolobium* make up >95% of trees on the black cotton clay soil savannas of East Africa and have been increasing in density since the 1970s (Niboye, 2010). These trees have a symbiotic relationship with several species of ants, providing domatia and food (Figure 1) in return for protection against herbivores (Hocking, 1970; Palmer et al., 2010). Fires in this region occur approximately every five years (i.e. prior to suppression; Heady, 1960), and both the ants and plants have adaptations to facilitate post‐fire recovery. Some ant species are able to detect smoke from up to two kilometers away and respond by evacuating to the soil or to holes within the trunk, where survival is enhanced (Cochard, 2004; Kimuyu et al., 2014; Sensenig et al., 2017). *Vachellia drepanolobium*, even when burned to the ground (top‐killed), typically resprout from the root crown and quickly produce thick foliage and domatia that can house their ant defenders (Okello et al., 2001, 2008), a pattern also observed in the related ant‐associated tree, *Vachellia zanzibarica* (Cochard et al., 2008). Quick resprouting could help *V*. *drepanolobium* outcompete other plants and speed recolonization by their ant symbionts, whose protection is potentially critical given that herbivores are attracted to new foliage in post‐burn areas (LaMalfa et al., 2019; Palmer et al., 2000; Zavala & Holdo, 2005).

For plant species capable of resprouting, the nutrient storage capacity of a large root system and deep taproot are key to surviving multiple top‐kills by fire before being able to grow tall enough to escape this “fire trap” (McCulley et al., 2004; Peguero & Espelta, 2011; Wigley et al., 2008). Many factors can affect the state of a plant's energy reserves. Nutrient limitation, leaf senescence in advance of a cold or dry season, and leaf flushing can all trigger plant‐wide shifts in nutrient transportation and storage (Chidumayo, 1994; Hermans et al., 2006; Kobe et al., 2005; Tolsma et al., 1987; Vergutz et al., 2012). Savanna plants demonstrate seasonal patterns of nutrient accumulation and translocation, with stores built up in the roots over the course of the rainy season and then diminished following recovery from dry season fires (Laclau et al., 2002; McIvor, 1981). On shorter time scales, many plants translocate nutrients – in particular, carbon from the leaves and nitrogen from the roots – in daily fluxes from sources to sinks (Ourry et al., 1996; Siebrecht et al., 2003). Thus, within the constraints of tissue capacities to act as sources and sinks, plant nutrient allocation is malleable, changing daily, seasonally, and under stressful conditions in response to a wide range of stimuli.

Plant biomass allocation (e.g. the ratio of root mass to shoot mass) can be influenced by the detection of volatiles released by neighboring plants (Ninkovic, 2003). Volatile communication is common in plants and can trigger a variety of responses. Competing plants release volatiles that inhibit cell growth and DNA synthesis (Nishida et al., 2005), while plants under attack from herbivores or pathogens emit compounds that elicit shifts in the direct and indirect defenses of others nearby, often within only a couple of days (reviewed in ref. Heil & Karban, 2010). One volatile stimulus that merits further study, especially for fire‐adapted plants such as *V*. *drepanolobium*, is smoke.

In advance of a fire, smoke can serve as a cue to seeds that the landscape will soon be newly cleared of competition and rich with nutrients, creating ideal germinating conditions (Allen et al., 1969; Kanz, 2001). Compounds isolated from smoke, such as the butanolide KAR1 (3‐methyl‐2*H*‐furo[2,3‐*c*]pyran‐2‐one) (Flematti et al., 2004; Keeley & Pausas, 2018; Van Staden et al., 2004), trigger seed germination in numerous fire‐adapted plants, including at least two *Vachellia* species – *V*. *robusta* and *V*. *hebeclada* (reviewed in ref. Kulkarni et al., 2007; Van Staden et al., 2000). In seedlings – plants between one week and 3 months old – studies have shown increased root and shoot growth in response to smoke treatments, both for species adapted to fire and those that are not (reviewed in ref. Light et al., 2009).

For established plants, unlike for seeds and seedlings, smoke is more likely a threatening cue that aboveground tissues will soon be destroyed and nutrients such as nitrogen volatilized (Chen et al., 2010; Cook, 1994). Yet the field of plant smoke response has been largely restricted to measuring seed germination rates and seedling growth and vigor. Research has focused on crop and non‐fire‐adapted species, and experimental protocols often employ an aqueous solution containing smoke compounds in lieu of smoke itself (e.g. reviews by Van Staden et al., 2000 and Light et al., 2009). In contrast to experiments examining volatile‐induced herbivore defenses (e.g. Baldwin & Schultz, 1983; Glinwood et al., 2009), to our knowledge no studies have explicitly tested smoke responses of older, more mature plants of fire‐adapted species and assessed their ability to respond physiologically to smoke volatiles.

We exposed *V*. *drepanolobium* of various ages to smoke: a 3‐month‐old cohort similar in age to past studies on smoke effects in other plants and two older cohorts of 9‐month‐old and 2‐year‐old trees. If, like their ant symbionts, the plants are able to detect smoke and recognize it as a sign of approaching fire (Sensenig et al., 2017), we hypothesized that the plants would respond by translocating nutrients away from vulnerable aboveground tissue into the insulated roots. By storing nutrients in their root system and preventing them from being lost to fire, *V*. *drepanolobium* would have the resources to quickly resprout following fire and thus rapidly reacquire its ant symbionts and increase its likelihood of escaping the fire trap (Clarke & Knox, 2009; LaMalfa et al., 2019; Schutz et al., 2009).

## 2 METHODS

### 2.1 Rearing, selection, and randomization

Seeds of *V*. *drepanolobium* were collected in the Kajiado North District of Kitengela, Kenya (36° 49’ E, 1° 23’ S, 1660 m elevation) and germinated at the Arnold Arboretum greenhouses in Boston, MA. Seeds were planted in Metro‐Mix® 852 soil in three different cohorts and grown into 3‐month‐old, 9‐month‐old, or 2‐year‐old plants. Experiments were conducted in late November 2018 with the 2‐year and 3‐month cohorts (planted July 2016 and August 2018, respectively) and again in December 2019 with the 9‐month cohort (planted Mach 2019). All plants were moved to the Harvard Museum of Comparative Zoology Laboratory greenhouse (6.3 miles due north from the Arnold Arboretum) for testing, with a period of at least a few weeks for acclimation to the new location. The 2‐year and 3‐month cohorts had thirty‐six plants each, and the 9‐month cohort had 50 plants. The plants were size‐matched within cohorts, using stem diameter as an index of overall size to reduce variation between the two treatment groups. Plants were then randomly assigned to a treatment protocol: smoke exposure or control. All plants were stored in the control greenhouse, which was kept closed to prevent stray smoke volatiles from entering, before they underwent their designated exposure and processing protocols.

### 2.2 Exposure protocol

One greenhouse was designated for controls and another for smoke exposure, each containing a tent within. The tents used were the Wakeman six‐person water‐resistant outdoors dome model, with dimensions of 10 ft by 10 ft at their base and 6 ft at their maximum internal height. These were made of 190T polyester with polyurethane coating, fiberglass structural poles, and a polyethylene floor shell. After unpacking, tents were initially aired out in full sunlight for several days before use. The tents served to reduce smoke dissipation over the course of the treatment period. Identical tents were assigned to either smoke or control treatments and consistently used for these treatments throughout.

One plant per treatment group was put inside a tent at a time. The smoke treatment tents were filled with smoke using a Breville wood smoke culinary infuser (BSM600SIL model, “The Smoking Gun”) and dried‐and‐mulched stem tissue obtained from pruning other *V*. *drepanolobium* in the greenhouse. We maintained the amount of smoke in the tents at a qualitatively consistent level through one to three repeated applications over the course of an hour. The two greenhouses, one with the smoking tents and the other with the controls, were separated by a hallway that was continuously exhausted by a construction‐site exhaust fan to prevent any residual smoke contamination. Researchers were kept constant between treatments to avoid exposing control plants to any residual smoke volatiles clinging to clothing. After one hour of exposure, both the control and the smoked plants were removed from their tents and left for 24 h in the same greenhouse room where they had undergone treatment. This time period was deemed sufficiently long for any fire‐related responses have taken place to their fullest extent, while soon enough for changes to still be detectable and not have reverted. The plants for that set of exposures were then removed from the greenhouse and immediately processed for nutrient analysis, using the processing protocol detailed below. During the entire experiment, the greenhouse temperature was maintained at about 26.7°C, simulating the natural environment of *V*. *drepanolobium*.

### 2.3 Processing and drying protocol

Post‐treatment, plants were processed manually, and each was sorted into leaves, stems, and roots. Aboveground and belowground tissues were cut apart, leaves were removed from stems, and roots were removed from soil and washed. Remaining water from washing was then absorbed using paper towels. The plant tissues were weighed to obtain the wet mass of each tissue type per plant. The root biomass was an underestimate due to non‐negligible quantities of root tissue lost during the soil washing stage. However, the amount lost is likely consistent across all plants within a cohort. The tissues were then enclosed in paper towel packets or glassine envelopes, dried at 40°C for around 24 h, and then weighed again to obtain the dry mass. The dried tissues were packaged and sent to an agricultural analysis company for nutrient data.

### 2.4 Plant nutrient analysis

The composition of plant macronutrients and micronutrients were analyzed by Spectrum Analytic (Washington Court House, Ohio), where samples were dried further, homogenized to a fine powder, and analyzed for composition of the following elements: nitrogen (N), phosphorus (P), potassium (K), calcium (Ca), magnesium (Mg), sulfur (S), boron (B), copper (Cu), iron (Fe), manganese (Mn), zinc (Zn) and sodium (Na). Spectrum Analytic reported high variability in the accuracy of Fe measurements; so these data were excluded from our analyses. In the case of the 2‐year plants from the first experiment in 2018, where sufficient tissue was available, percent carbon (C) was also analyzed. Elemental composition was expressed in the form of percent total dry mass by weight for macronutrients and parts per million for micronutrients. We derived the total mass of each element per plant by summing leaf and root nutrient concentrations. In the case of the 3‐month seedlings, only 10 individuals per treatment group had sufficient tissue for nutrient analyses, and there was insufficient dry mass of root tissue for nutrient analyses beyond percent nitrogen. Similarly, for the two younger cohorts, insufficient quantities of stem tissue precluded accurate nutrient measurements; thus, this tissue was excluded from our analyses.

### 2.5 Data analysis

For each of the three age cohorts (2‐year, 9‐month, and 3‐month) and for each tissue (leaf and root), Welch's two‐sample *t*‐test was used to compare nutrient concentrations between smoke treatment and control. The Benjamini‐Hochberg correction for multiple testing was used to determine significance, with a false discovery rate of *Q *= 0.05 and *m *= the number of tests (Benjamini & Hochberg, 1995).

To examine differences in baseline conditions between each of the age cohorts, control plant leaf and root nutrient concentrations were summed to obtain a measure of whole‐plant nutrition. Dry masses of the tissues were summed to obtain total mass, and root‐shoot ratios were calculated by dividing the dry mass of root tissue by the dry mass of leaf tissue for each plant. Pairwise comparisons between the 2‐year and 9‐month cohorts were made using Welch's *t*‐test and by calculating fold change (Δfold). Due to the small amount of tissue available from the 3‐month cohort, only total mass, root‐shoot ratio, and total nitrogen could be calculated. For these three measures, additional pairwise comparisons were made with the 9‐month cohort.

To investigate the possibility that changes in tissue nutrient concentration were due to altered photosynthetic or root uptake rates, rather than translocation, whole‐plant properties were also compared between smoke and control cohorts within each cohort. Welch's *t*‐test was used to determine whether whole‐plant nutrient concentrations differed between treatments and thus whether an external nutrient source or sink should be considered.

All statistical analyses were performed using R Version 4.0.0 (Hunter, 2007; Kassambara, 2020; R Core Team, 2020). Violin plots were created using the R packages ‘ggplot2’ (Wickham, 2016) and ‘ggsignif’ (Ahlmann‐Eltze, 2019).

## 3 RESULTS

### 3.1 Tissue nutrient concentrations

**FIGURE 2:**
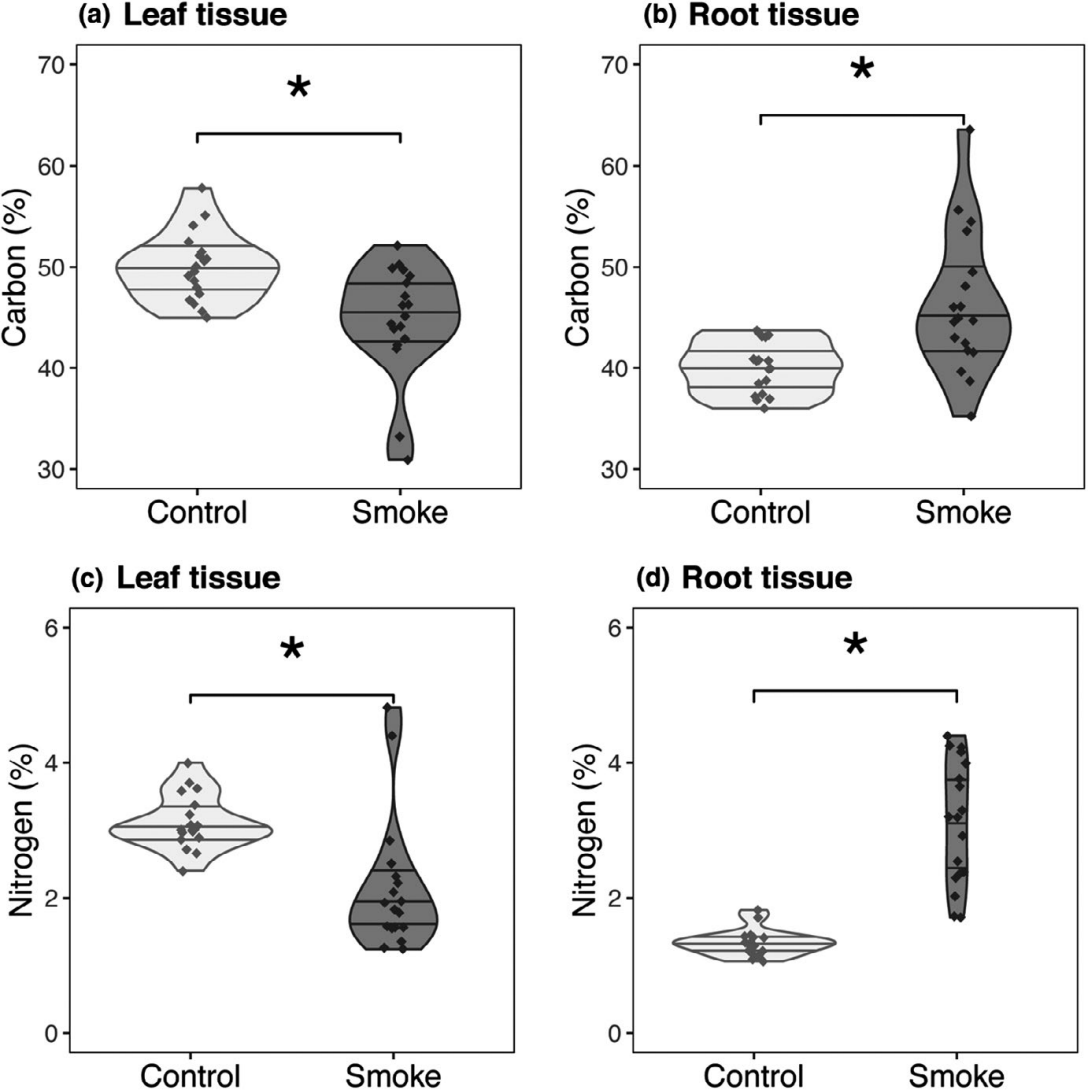
Comparison of tissue nutrient concentrations between smoke‐exposed and control 2‐year‐old plants. Nutrient concentrations were measured in leaf tissue (a, c) and root tissue (b, d). Sample size per treatment was *n* = 18 plants. Control plants are shown in light gray, smoke‐exposed plants in dark gray. All percentages are percent masses. Asterisks indicate significant differences between treatments, using the Benjamini‐Hochberg correction for multiple testing (*Q* = 0.05, *m *= 13, significance cut‐off of *P *= .003). Nutrient analyses were performed by Spectrum Analytic

The carbon and nitrogen concentration in tissues differed significantly between control and smoke‐exposed 2‐year *V*. *drepanolobium* in a manner consistent with adaptive translocation in response to impending fire (Table 1). For the 2‐year cohort, smoke‐exposed plants had 10% lower leaf carbon (*t *= 3.33, *p* < .001) and 16% higher root carbon (*t *= −3.58, *p* < .001) than controls. The 2‐year‐old plants had 31% lower leaf nitrogen (*t *= 3.82, *p* < .001) and 126% higher root nitrogen (*t *= −8.12, *p* < .001) than controls (Figure 2). By contrast, the smoke‐exposed 3‐month‐old plants 14% lower root nitrogen (*t *= 4.44, *p* < .001) than controls (Table 1). This cohort also had a non‐significant trend of 6% higher leaf nitrogen (*t *= −1.98, n.s., *p* = .07), an opposing pattern from that observed in the 2‐year‐old plants. The 9‐month‐old plants did not show significant tissue nutrient differences between treatments (Table 1).

**TABLE 1:** Comparison of leaf and root nutrient content of smoke‐exposed and control *Vachellia drepanolobium* for each of the three age cohorts

Age cohort	2‐year‐old		9‐month‐old		3‐month‐old	
Tissue	Leaf (*n* = 18)	Root (*n* = 18)	Leaf (*n* = 22)	Root (*n* = 19)	Leaf (*n* = 18)	Root (*n* = 10)
Nutrient	*t*‐value (*p*)	*t*‐value (*p*)	*t*‐value (*p*)	*t*‐value (*p*)	*t*‐value (*p*)	*t*‐value (*p*)
C (%)	3.33* (<.001)	−3.58* (.002)	–	–	–	–
N (%)	3.82* (<.001)	−8.12* (<.001)	−0.04 (.68)	−0.17 (.86)	−1.98 (.07)	4.44* (<.001)
P (%)	−0.57 (.57)	−2.43 (.02)	2.36 (.02)	1.39 (.17)	−1.18 (.26)	–
K (%)	−0.04 (.97)	−1.53 (.14)	−0.24 (.81)	−0.30 (.76)	−0.64 (.53)	–
Ca (%)	−0.73 (.47)	−2.08 (.05)	−0.92 (.37)	0.46 (.65)	−2.89 (.01)	–
Mg (%)	−2.09 (.05)	−2.18 (.04)	−1.01 (.32)	−1.76 (.09)	0.01 (.99)	–
S (%)	0.57 (.57)	−0.30 (.76)	−0.25 (.81)	0.01 (.99)	−2.18 (.05)	–
Mn (ppm)	−1.12 (.27)	−0.83 (.42)	−0.73 (.47)	−0.56 (.58)	0.04 (.97)	–
B (ppm)	0.13 (.90)	−4.40* (<.001)	0.28 (.78)	0.39 (.70)	−1.77 (.10)	–
Zn (ppm)	−0.72 (.48)	−2.33 (.03)	0.84 (.41)	−1.42 (.17)	−0.07 (.95)	–
Cu (ppm)	−0.03 (.98)	−0.43 (.67)	−0.34 (.74)	−1.57 (.13)	0.07 (.95)	–
Na (ppm)	−1.33 (.19)	−0.19 (.85)	−0.45 (.66)	−0.32 (.75)	1.50 (.16)	–

Note

The number of plants per treatment (*n*) varied between tissues when some samples were not viable for nutrient analysis. Carbon percentage was only measured for the 2‐year cohort. All percentages are percent masses. Welch's *t* test was used to compare between treatments; positive *t*‐values signify nutrient concentration was higher in the control group, and negative t‐values signify it was higher in the smoke‐exposed group. Significance was calculated using the Benjamini‐Hochberg correction for multiple testing (*m* = 13 for 2‐year cohort and *m* = 12 for the 3‐month and 9‐month cohorts, *Q* = 0.05), leading to an initial *p*‐value significance cut‐off of .003. Significant differences are shown by bolded and asterisked values. The 3‐month‐old plants had insufficient root tissue to measure any nutrient other than nitrogen. Nutrient analyses were performed by Spectrum Analytic.

In the 2‐year cohort, boron was 21% higher in the root tissue of smoke‐exposed plants (*t *= −4.398, *p* < .001), but not correspondingly lower in the leaves (*t *= 0.13, n.s., *p* = .90). There were non‐significant trends toward higher magnesium in both the leaves (*t *= −2.18, n.s., *p* = .04) and the roots (*t *= −2.086, n.s., *p* = .05) of smoke‐exposed plants (Table 1). No other nutrients showed significant differences between treatments, and no other significant differences were found for the 9‐month and 3‐month cohorts (Table 1).

### 3.2 Whole‐plant differences

For control plants, total dry biomass increased significantly with age, from 3 months old (0.89 g) to 9 months old (11.1 g) to 2 years old (28.3 g; Table A1). The average ratio of root mass to shoot mass (mean root–shoot ratio) of 2‐year‐old plants was 6.25, significantly larger than that of the two younger cohorts (0.85 for 9‐month and 0.81 for 3‐month) (Table A1). There were no strong correlations between root–shoot ratio and total mass or total nutrient concentration (all *r* ^2^ < 0.1). The younger cohorts had higher concentrations of most nutrients in their tissues, with the exception of sodium and calcium, which were higher in the 2‐year‐old plants, and boron and zinc, which showed no significant differences between cohorts (Table A1).

Within each age cohort, whole‐plant (leaf plus root) nutrient concentration did not differ significantly between treatments after correcting for multiple testing (Table A2). However, the 2‐year‐old smoke‐exposed plants showed non‐significant trends toward higher total nitrogen (*t *= −2.87, n.s., *p *= .009) and magnesium (*t *= −2.99, n.s., *p *= .006) than the controls (Table A2).

## 4 DISCUSSION

These results provide novel evidence that 2‐year‐old *V*. *drepanolobium* respond to smoke by translocating carbon and nitrogen from the leaves to the roots. Shoot‐to‐root translocation of macronutrients may reduce nutrient losses to fire and aid resprouting of top‐killed *V*. *drepanolobium*. Thus, this behavior could represent an inducible fire adaptation in plants. Future research should address the timescale over which this response occurs, beyond our finding that such a difference was measurable 24 h after smoke exposure. Due to the destructive nature of collecting tissues for nutrient analyses, time series data were not feasible for these experiments. Techniques involving spectroscopy are a promising possibility for monitoring changes in leaf tissue nitrogen concentrations over time, thus elucidating both the speed of the response and its duration following smoke exposure (e.g. Blackmer et al., 1994).

The purpose of translocation may differ between nutrients. Nitrogen‐fixing plants like *Vachellia* are known to trade sugars to symbiotic rhizobial bacteria and mycorrhizal fungi in exchange for nutrients like nitrogen and phosphorus, which can be limiting in savanna ecosystems (Ahiabor et al., 2007; Dodd et al., 1990; Sanginga et al., 1996). Carbon translocation, in the form of sugars, could fuel this interaction in anticipation of regrowth, while nitrogen translocation would reduce its loss to volatilization and the subsequent fixation needs. Boron and magnesium, which showed clearly directional trends in response to smoke, are in turn important nutrients for sugar translocation (Gauch & Dugger, 1953; Hermans & Verbruggen, 2005). Anticipatory nutrient translocation may elucidate past findings in a South African savanna showing that plant nutrient stoichiometry and composition were surprisingly resilient to fire and the associated changes in soil nutrient distribution (Pellegrini et al., 2015).

The remaining nutrients showed no significant translocation in the 2‐year cohort, which could reflect their integration into unalterable tissues and structures, low phloem mobility (Bukovac & Wittwer, 1957), or a translocation timescale different from 24 h. Seasonal translocation patterns are known to vary between nutrients for different savanna species (Tolsma et al., 1987). There were also no significant shifts in whole‐plant nutrient concentrations between the smoke and control treatments for any age cohort. This suggests no changes in photosynthetic rate or root nutrient uptake occurred in response to smoke (Table A2).

The youngest cohort of *V*. *drepanolobium* showed the opposite pattern of smoke‐induced translocation from the oldest cohort. While small tissue quantities prevented the analysis of most of the nutrients, nitrogen was significantly lower in the roots and non‐significantly higher in the leaves, consistent with its movement from belowground to aboveground tissues in these 3‐month‐old plants. Previous studies have found that newly germinated seedlings respond to volatile compounds present in smoke with increased root and shoot growth (reviewed in Light et al., 2009). If, like in related *Vachellia* species, smoke is a cue to germinate and grow, these 3‐month‐old plants may be exhibiting a residual form of this response. Furthermore, small *V*. *drepanolobium* are much more likely to die in a fire than larger individuals (Okello et al., 2008), undermining any selective pressure for young plants to translocate nutrients belowground in anticipation of resprouting.

We did not find significant nutrient translocation in the 9‐month‐old plants. This may reflect an intermediate point in the ontogeny of nutrient translocation responses, lying somewhere between the root‐to‐shoot response of the 3‐month‐old plants and the shoot‐to‐root response of the 2‐year‐old plants. A developmental shift could relate to reproduction, as the 2‐year cohort were flowering, while the younger cohorts appeared to be pre‐reproductive. Alternatively, a large root–shoot ratio could be necessary for shoot‐to‐root translocation, acting as a sink for drawing down nutrients from the leaves. The 9‐month cohort, despite having around ten times the mass of the 3‐month cohort, still had an average root–shoot ratio of less than 1.0 (Table A1). Without a substantial belowground sink, the plant may be unable to form a gradient to translocate nutrients. Finally, although the 2‐year‐old plants had higher concentrations of sodium and calcium, which can influence translocation capacity (e.g. Gauch & Dugger Jr., 1953; Subbarao et al., 2003), the younger cohorts had higher nutrient concentrations generally (Table A1), and this may have modulated their root–shoot development as well as their ability to send nutrients to the roots (Ericsson, 1995). In studies of nutrient withdrawal from senescing leaves, trees growing in nutrient‐rich environments had reduced translocation efficiency compared to those growing in nutrient‐poor environments (Kobe et al., 2005; Vergutz et al., 2012).

This study provides a first steppingstone toward understanding how older plants use smoke as a cue for fire‐adaptive physiological shifts, and how these responses might vary during development. On the organismal level, our findings raise questions about the mechanisms and timing of smoke‐induced nutrient translocation in *V*. *drepanolobium* and motivate a search for related responses in other fire‐adapted species. At a community level, symbiotic ants and soil microbial and fungal communities could influence and be influenced by this response, highlighting the need for field‐based experiments. Seasonality, rainfall, and natural gradients in soil nutrients across the landscape also likely interact with and modulate plant nutrient translocation. On the ecosystem level, given the changing density of *V*. *drepanolobium* on East African savannas, accounting for these findings may alter predictions of how landscape‐scale nutrient flows will respond to anthropogenic fire management or woody encroachment. Much remains to be discovered of inducible smoke responses in plants beyond the seed and young seedling stages.

## CONFLICT OF INTEREST

None declared.

## AUTHOR CONTRIBUTION

**Richard Rabideau‐Childers:** Conceptualization (equal); Data curation (equal); Formal analysis (equal); Funding acquisition (equal); Investigation (equal); Methodology (equal); Supervision (equal); Writing‐original draft (equal); Writing‐review & editing (equal). **Katherine I. W. Angier:** Conceptualization (equal); Data curation (equal); Formal analysis (equal); Funding acquisition (equal); Investigation (equal); Methodology (equal); Writing‐original draft (equal); Writing‐review & editing (equal). **Brendan Z. M. Dean:** Conceptualization (equal); Data curation (equal); Formal analysis (equal); Funding acquisition (equal); Investigation (equal); Methodology (equal); Writing‐original draft (equal). **Meghan Blumstein:** Data curation (equal); Formal analysis (equal); Investigation (equal); Methodology (equal); Resources (equal); Writing‐review & editing (equal). **Walker S. Darling:** Data curation (equal); Formal analysis (equal); Investigation (equal); Methodology (equal); Writing‐review & editing (equal). **Annina Kennedy‐Yoon:** Data curation (equal); Formal analysis (equal); Investigation (equal); Methodology (equal); Writing‐review & editing (equal). **Clayton H. Ziemke:** Data curation (equal); Formal analysis (equal); Investigation (equal); Methodology (equal); Writing‐review & editing (equal). **Christian A. Perez‐Martinez:** Data curation (equal); Formal analysis (equal); Investigation (equal); Methodology (equal); Writing‐review & editing (equal). **Donghao Wu:** Investigation (equal); Writing‐review & editing (equal). **Wenqing Ye:** Investigation (equal); Writing‐review & editing (equal). **Inam Yekwayo:** Data curation (equal); Formal analysis (equal); Investigation (equal); Methodology (equal); Writing‐review & editing (equal). **Duncan M. Kimuyu:** Conceptualization (equal); Investigation (equal); Methodology (equal); Project administration (equal); Resources (equal); Supervision (equal); Writing‐review & editing (equal). **Dino J. Martins:** Conceptualization (equal); Investigation (equal); Methodology (equal); Project administration (equal); Resources (equal); Supervision (equal); Writing‐review & editing (equal). **Naomi E. Pierce:** Conceptualization (equal); Formal analysis (equal); Funding acquisition (equal); Investigation (equal); Methodology (equal); Project administration (equal); Resources (equal); Supervision (equal); Writing‐original draft (equal); Writing‐review & editing (equal).

## Data Availability

Nutrient data can be found at Dryad https://doi.org/10.5061/dryad.b5mkkwhdj.

## APPENDIX 1

**TABLE A1:** Comparison between age cohorts of whole‐plant properties in control plants

Variable	2‐year (*n* = 18)	9‐month (*n* = 19)	Δfold	*t*‐value (*p*)	3‐month (*n *= 10)	*t*‐value (*p*)
Mass (*g*)	28.3	11.09	**2.6**	**5.56* (<.001)**	0.89	**10.81* (<.001)**
Root/shoot	6.25	0.85	**7.4**	**6.32* (<.001)**	0.81	0.30* (.767)
N (%)	4.46	7.18	**0.6**	**−17.16* (<.001)**	6.57	**3.35* (.003)**
P (%)	0.35	1.25	**0.3**	**−17.26* (<.001)**	–	–
K (%)	2.36	4.52	**0.5**	**−11.56* (<.001)**	–	–
Ca (%)	3.47	2.74	**1.3**	**4.11* (<.001)**	–	–
Mg (%)	0.47	0.89	**0.5**	**−17.99* (<.001)**	–	–
S (%)	0.62	0.92	**0.7**	**−9.00* (<.001)**	–	–
Mn (ppm)	135	181.79	**0.7**	**−2.76* (.001)**	–	–
B (ppm)	111.06	104.25	1.1	0.94 (.021)	–	–
Zn (ppm)	90.78	104.57	0.9	−1.88 (.188)	–	–
Cu (ppm)	17.27	26.88	**0.6**	**−5.18* (<.001)**	–	–
Na (ppm)	7331.67	1664.32	**4.4**	**11.77* (<.001)**	–	–

Note

Leaf and root nutrient concentrations were summed and compared between 2‐year, 9‐month, and 3‐month control cohorts. Welch’s *t*‐tests were used for pairwise comparisons of the 2‐year and 9‐month cohorts, and additional pairwise tests were run between the 9‐month and 3‐month cohorts for mass, root–shoot ratio, and nitrogen. The Benjamini‐Hochberg correction for multiple testing was used to determine significance (*Q *= 0.05, *m *= 13), leading to an initial *p*‐value significance cut‐off of .003. Fold change (Δfold) compares the 2‐year and 9‐month cohorts; Δfold >1 signifies a greater value for the 2‐year cohort, while Δfold <1 signifies a greater value for the 9‐month cohort. Significant differences are shown by bolded and asterisked values. Nutrient analyses were performed by Spectrum Analytic.

**TABLE A2:** Comparison of whole‐plant properties between smoke‐exposed and control trees for each age cohort

Age cohort	2‐year‐old (*n* = 18)	9‐month‐old (*n *= 19)	3‐month‐old (*n *= 10)
	*t*‐value (*p*)	*t*‐value (*p*)	*t*‐value (*p*)
Mass (g)	0.61 (.55)	1.15 (.26)	2.48 (.02)
Root/shoot	1.28 (.21)	0.25 (.81)	0.46 (.65)
C (%)	0.55 (.59)	–	–
N (%)	−2.87 (.009)	−0.02 (.99)	0.46 (.65)
P (%)	−1.97 (.06)	2.36 (.025)	–
K (%)	−0.52 (.61)	−0.38 (.71)	–
Ca (%)	−1.55 (.13)	−0.76 (.45)	–
Mg (%)	−2.99 (.006)	−1.53 (.14)	–
S (%)	0.21 (.84)	−0.17 (.87)	–
Mn (ppm)	−1.14 (.26)	−1.08 (.29)	–
B (ppm)	−0.47 (.64)	0.48 (.64)	–
Zn (ppm)	−2.04 (.05)	−1.17 (.26)	–
Cu (ppm)	−0.25 (.80)	−1.90 (.07)	–
Na (ppm)	−1.09 (.28)	−0.44 (.66)	–

Note

There were *n* individual plants per treatment (smoke or control). Nutrients shown are the sum of leaf and root concentrations within each treatment group. All percentages are percent masses. Carbon (C) was only measured for the 2‐year‐old plants. Due to the small mass in the 3‐month cohort, whole‐plant concentrations could not be calculated beyond nitrogen. Welch’s *t*‐test was used to compare between experimental treatments. The Benjamini‐Hochberg procedure was used to determine significance (*Q* = 0.05, *m *= 14 for 2‐year, *m *= 13 for 9‐month), leading to a significance cut‐off of 0.003. No significant differences were found. Nutrient analyses were performed by Spectrum Analytic.
